# Use of social network analysis and global sensitivity and uncertainty analyses to better understand an influenza outbreak

**DOI:** 10.18632/oncotarget.15076

**Published:** 2017-02-03

**Authors:** Jianhua Liu, Hongbo Jiang, Hao Zhang, Chun Guo, Lei Wang, Jing Yang, Shaofa Nie

**Affiliations:** ^1^ Department of Epidemiology and Biostatistics, School of Public Health, Tongji Medical College, Huazhong University of Science and Technology, Wuhan, Hubei, China; ^2^ Department of Epidemiology and Biostatistics, School of Public Health, Guangdong Pharmaceutical University, Guangzhou, Guangdong, China; ^3^ Department of Infectious Diseases, Center for Disease Control and Prevention, Yichang City, Hubei, China

**Keywords:** field epidemiology, control of infectious diseases, social network analysis, global sensitivity and uncertainty analyses

## Abstract

In the summer of 2014, an influenza A(H3N2) outbreak occurred in Yichang city, Hubei province, China. A retrospective study was conducted to collect and interpret hospital and epidemiological data on it using social network analysis and global sensitivity and uncertainty analyses. Results for degree (*χ*^2^=17.6619, *P*<0.0001) and betweenness(*χ*^2^=21.4186, *P*<0.0001) centrality suggested that the selection of sampling objects were different between traditional epidemiological methods and newer statistical approaches. Clique and network diagrams demonstrated that the outbreak actually consisted of two independent transmission networks. Sensitivity analysis showed that the contact coefficient (*k*) was the most important factor in the dynamic model. Using uncertainty analysis, we were able to better understand the properties and variations over space and time on the outbreak. We concluded that use of newer approaches were significantly more efficient for managing and controlling infectious diseases outbreaks, as well as saving time and public health resources, and could be widely applied on similar local outbreaks.

## INTRODUCTION

Public health events occurred frequently in China. Take 2013 as an example, a total of 1,077 public health emergencies occurred. [1] Analysis of these emergencies only focused on traditional epidemiological methods in the past. Field epidemiological investigation skill has been identified as one of the five top weaknesses in national health emergency response skills and techniques. [2] Traditional epidemiological methods do not consistently provide reliable evidence on how to objectively identify the correct patients, how to select correct sampling objects for laboratory tests, and how to understand and describe outbreak characteristics. [3, 4] Social network analysis (SNA) and global sensitivity and uncertainty analyses (GSUA) are relatively new tools that can be used to address these problems. [5, 6] Using SNA, nodes and ties represent patients and connections between them in network diagrams. Through centrality analysis and connectedness measurement, important patients could be identified, and the propagation of outbreaks could be more accurately described. [7–10] By using these network analysis and graphics, SNA could be used to study outbreak structures and characteristics. [5, 11, 12] GSUA is the study of how uncertainties in the output of a model can be apportioned to different sources of uncertainty among model inputs. It is a variance-based method for analyzing data and models using an objective function. [6, 13, 14] GSUA can be used to rank parameters such as infection coefficient, contact coefficient, recovery rate and death rate based on their relative influence on the dynamics of simulated epidemics. [15] It can also inform researchers on the dynamics of investigation processes, and can potentially play an important role in outbreak management.

From Wednesday, July 16, 2014, to Monday, August 4, 2014, an influenza A(H3N2) outbreak with 63 cases, including nine laboratory-confirmed positive cases, occurred in an isolated compulsory detoxification center. Yichang center for disease control and prevention managed the outbreak using traditional and molecular epidemiological methods, and reported it as a general public health emergency (grade IV) in the China Information System for Disease Control and Prevention.

## RESULTS

### Selection of sampling objects

Normalized centrality measures (degree and betweenness) of all 72 index cases were analyzed using SNA (Table 1). A total of 14 nodes (six from platoon A; eight from platoon B), all with high values of degree and betweenness centrality, were suggested to be sampling objects (Tables 2 and 3). Kruskal-Wallis test results showed statistical significance in degree centrality (*χ*^2^ = 17.6619, *P* < 0.0001, Table 2) and betweenness centrality (*χ*^2^ = 21.4186, *P* < 0.0001, Table 3), showing the two selection methods (SNA approach and traditional method) of sampling objects to be significantly different.

**Table 1:** Statistics of normalized centrality measures of all 72 index cases

Nodes	Degree	Between-ness	Nodes	Degree	Between-ness	Nodes	Degree	Between-ness
WXJ	16.901	4.547	WL^(a)^	2.817	0.040	XYE	2.817	0.523
FY^(b,c)^	1.408	0.000	YQ	1.408	0.000	TY	1.408	0.000
MHS^(a)^	1.408	0.000	XMM	1.408	0.000	YSB	1.408	0.000
CZM^(a)^	5.634	1.690	LD^(a)^	1.408	0.000	ZJH	1.408	0.000
DZM	1.408	0.000	CB	1.408	0.000	XL	1.408	0.000
MY	2.817	0.604	LJM	1.408	0.000	ZD^(a)^	1.408	0.000
JFENG	1.408	0.000	LG	1.408	0.000	WXK	2.817	0.523
BBC	1.408	0.000	FXP^(c)^	1.408	0.000	LLY	1.408	0.000
JFEI	1.408	0.000	YYJ^(c)^	1.408	0.000	ZL	1.408	0.000
ZNY	1.408	0.000	LXY	0.000	0.000	DP^(a)^	1.408	0.000
CXY	1.408	0.000	XQH	0.000	0.000	HWM	1.408	0.000
FHD	1.408	0.000	AJ	0.000	0.000	ZC	2.817	0.040
LBB	1.408	0.000	WJ	0.000	0.000	LZ	1.408	0.000
LJW	1.408	0.000	ZY	0.000	0.000	HK	1.408	0.000
MYQ^(a)^	1.408	0.000	TJ^(c)^	0.000	0.000	FJJ	2.817	0.040
CYF	1.408	0.000	LL^(c)^	0.000	0.000	FXZ	1.408	0.000
ZB	1.408	0.000	XQ^(c)^	0.000	0.000	QK	1.408	0.000
ZR^(b)^	1.408	0.000	CDY^(c)^	0.000	0.000	LCY	0.000	0.000
RHT^(a)^	4.225	0.362	WM	0.000	0.000	ZDY	0.000	0.000
ZYL	5.634	0.483	PJ^(c)^	0.000	0.000	QSQ	0.000	0.000
YW	1.408	0.000	XYY	12.676	3.421	HP	0.000	0.000
LY^(a)^	1.408	0.000	QHW	2.817	0.966	WP	0.000	0.000
ZJ	1.408	0.000	CJP	2.817	0.523	RH	0.000	0.000
GC	1.408	0.000	JHW	2.817	0.523	HZX^(b,c)^	0.000	0.000

(a): laboratory test positive

(b): laboratory test negative

(c): excluded cases

**Table 2:** Comparison of the degree of sampling objects in actual outbreak and our retrospective study suggestion

Nodes	Degree (actual)	Nodes	Degree (suggested)	Kruskal-Wallis Test	
FY	1.408	WXJ	16.901	Chi-Square	17.6619
MHS	1.408	CZM	5.634	DF	1
CZM	5.634	MY	2.817	Pr > Chi-Square	<.0001
ZNY	1.408	RHT	4.225		
MYQ	1.408	ZYL	5.634		
ZB	1.408	WL	2.817		
ZR	1.408	XYY	12.676		
RHT	4.225	QHW	2.817		
LY	1.408	CJP	2.817		
ZJ	1.408	JHW	2.817		
GC	1.408	XYE	2.817		
WL	2.817	WXK	2.817		
LD	1.408	ZC	2.817		
FXP	1.408	FJJ	2.817		
TJ	0.000				
LL	0.000				
XQ	0.000				
ZD	1.408				
DP	1.408				
HZX	0.000				
median	1.408		2.817		
Q1	1.408		2.817		
Q3	1.408		5.282		

**Table 3:** Comparison of the betweenness of sampling objects in actual outbreak and our retrospective study suggestion

Nodes	Betweenness (actual)	Nodes	Betweennes (suggested)	Kruskal-Wallis Test	
FY	0.000	WXJ	4.547	Chi-Square	21.4186
MHS	0.000	CZM	1.690	DF	1
CZM	1.690	MY	0.604	Pr > Chi-Square	<.0001
ZNY	0.000	RHT	0.362		
MYQ	0.000	ZYL	0.483		
ZB	0.000	WL	0.040		
ZR	0.000	XYY	3.421		
RHT	0.362	QHW	0.966		
LY	0.000	CJP	0.523		
ZJ	0.000	JHW	0.523		
GC	0.000	XYE	0.523		
WL	0.040	WXK	0.523		
LD	0.000	ZC	0.040		
FXP	0.000	FJJ	0.040		
TJ	0.000				
LL	0.000				
XQ	0.000				
ZD	0.000				
DP	0.000				
HZX	0.000				
Median	0.000		0.523		
Q1	0.000		0.392		
Q3	0.000		0.876		

### Outbreak characteristics

**Figure 1:**
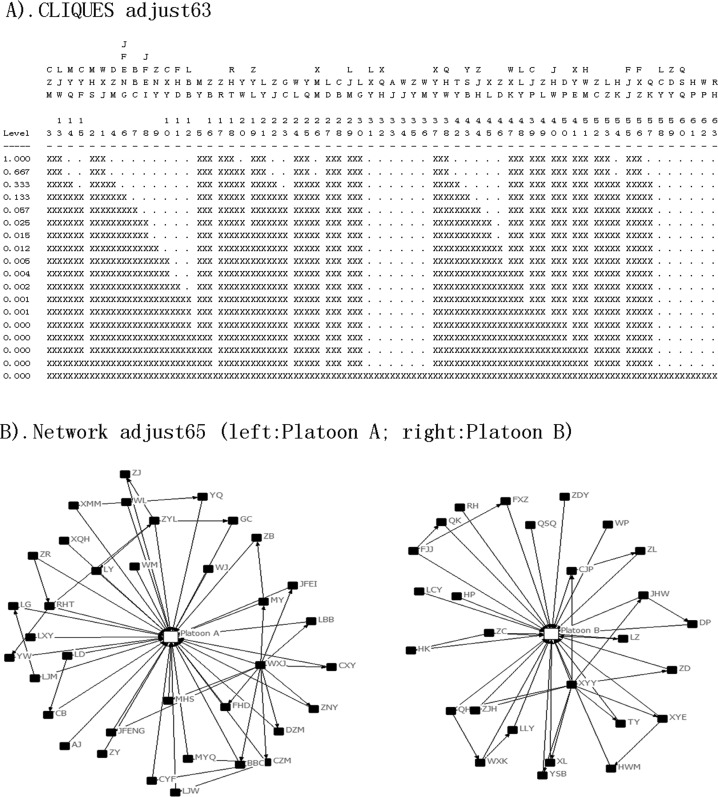
Diagrams of cliques and network structure, in an isolated compulsory detoxification center in Yichang City, Hubei Province, China, July to August 2014. A The two clusters of the matrix Adjust63. **B**. The two independent structures of the networks.

**Figure 2:**
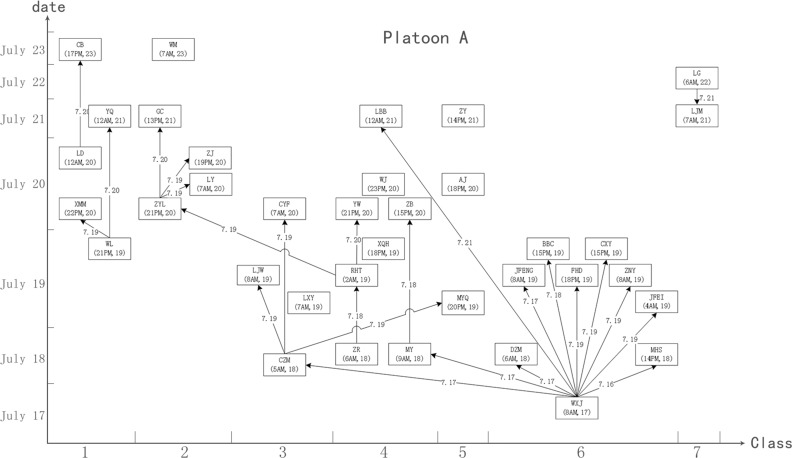
Schematic diagram demonstrate the possible propagation chain of platoon A The patient’s name and date of onset (accurate to hours) are shown in the box. The possible route and date of propagation are represented as an arrow. The *X* axis represents the onset date. The *Y* axis represents the classes (dormitories) where the patients lived. WXJ was the key node among the 36 patients, and 7 of the 9 classes involved were in platoon A.

**Figure 3:**
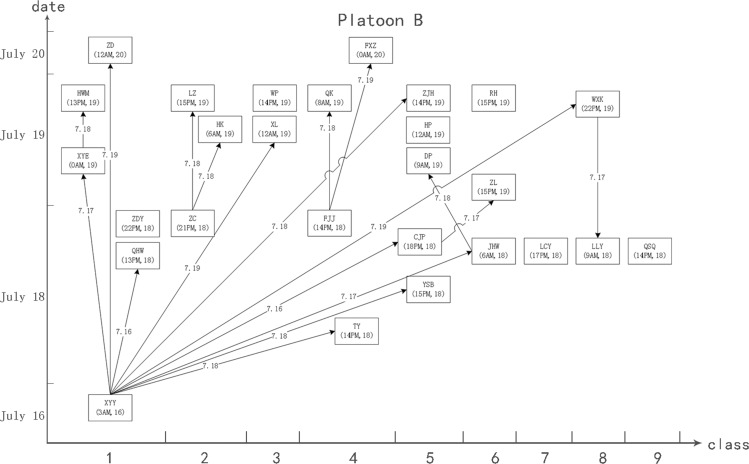
Schematic diagram demonstrate the possible propagation chain of platoon B The patient’s name and date of onset (accurate to hours) are shown in the box. The possible route and date of propagation are represented as an arrow. The *X* axis represents the onset date. The *Y* axis represents the classes (dormitories) where the patients lived. XYY was the key node among the 27 patients, and all 9 classes in platoon B were involved.

A total of 134 drug abstainers were strictly separated into two platoons; the 75 abstainers in platoon A resided on the second floor, and the other 59 abstainers in platoon B resided on the third floor. Each floor had its own workshop. The assigned exercise, work-break and dining areas were also separate. The mealtime of platoon A was 5 minutes earlier than that of platoon B. Therefore, there was no close contact between the two platoons. The resulting cliques also suggested that the epidemic situation may have contained two or more networks. Figure 1 demonstrates the cliques and structure of the networks. Figures 2 and 3 shows the schematic diagrams of the possible propagation chain of the two platoons.

Platoon A, which had 36 patients and 25 pairs of close contacts, was divided into eight classes. Class 1 consisted of 11 drug abstainers with five patients (represented as 11(5)). Similarly, classes 2 to 8 had compositions of 12(5), 11(4), 10(8), 10(3), 10(9), 6(2), and 5(0) abstainers and patients, respectively. The first case (patient WXJ) was reported on July 17, and there were 5, 12, 10, 5, 1 and 2 cases reported on each subsequent day thereafter. Platoon B had 27 patients with 18 pairs of close contacts and was divided into nine classes. Classes 1 to 9 contained 8(6), 8(3), 8(2), 6(4), 6(5), 7(3), 7(1), 5(2), and 4(1) abstainers and patients, respectively. The first case (patient XYY) was reported on July 16, and there were 11 cases reported on July 18, 13 cases on July 19, and two cases on July 20.

A total of 21 contacts were recorded in the dormitory, six in the refectory and eight in other places from platoon A, and 16 in the dormitory, three in the refectory and four in other places from platoon B ($\chi^{2}cMH$ = 0.5403, *P* = 0.7633). A total of 31 direct and six indirect contacts were found in platoon A, whereas 20 direct and 10 indirect contacts were found in platoon B. More direct contacts than indirect contacts were found among the patients, although the difference (*χ*^2^ = 2.6704, *P* = 0.1022) was not statistically significant.

### Global sensitivity and uncertainty analyses

Factor *k* (contact coefficient) was distributed as three different ranges. When distributed as *k* ~ beta (2, 7), the first-order indices of factors *v*, *k* and *r* were 0.4144, 0.4450, and 0.0002, respectively, and the total-order indices were 0.5549, 0.5855, and 0.0001, respectively. Other indices are shown in Table 4.

**Figure 4:**
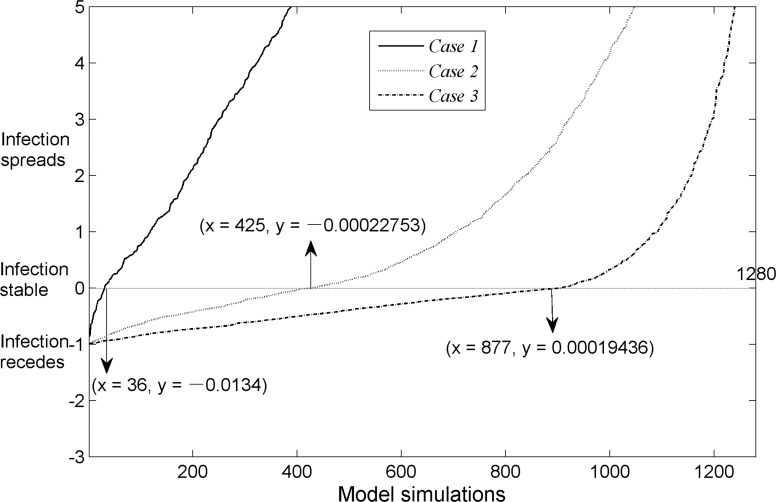
Uncertainty analysis shows infection dynamics for the three cases Dynamic change of infection rate is plotted on the *Y* axis (positive *Y* indicates infection propagation, negative *Y* indicates subsidence) by the output variable of interest. The plot shows that the critical values for cases 1-3 are 36, 425 and 877, respectively. The *X* axis represents model runs (total of 1,280). The model outputs for each case are sorted in ascending order, so that each plot is a monotonic curve, and the *Y* axis is cut (scaled from -3 to +5) to visualize the plot around zero.

Figure 4 shows three cases of the infection dynamics exercise based on uncertainty analysis. The plot shows that the infection propagated in 97.19% (case 1), 66.80% (case 2), and 31.56% (case 3) of individuals in each case, respectively.

**Table 4:** First-order and total-order indices generated by sensitivity analysis

Factors	*k*~beta(2,7)		*k*~beta(0.5,10)		*k*~beta(0.2,15)	
	First-order indices	Total-order Indices	First-order indices	Total-order Indices	First-order indices	Total-order Indices
Infection(*v*)	0.4144 (48.21%)	0.5549 (48.65%)	0.1294 (16.03%)	0.3277 (27.35%)	0.0588 (7.57%)	0.2910 (23.62%)
Contact(*k*)	0.4450 (51.77%)	0.5855 (51.34%)	0.6689 (82.84%)	0.8671 (72.36%)	0.6940 (89.38%)	0.9261 (75.16%)
Recovery(*r*)	0.0002 (0.02%)	0.0001 (0.01%)	0.0091 (1.13%)	0.0035 (0.29%)	0.0237 (3.05%)	0.0150 (1.22%)
Total	0.8596	1.1405	0.8074	1.1983	0.7765	1.2321

## DISCUSSION

Following the principles of efficiency and effectiveness, investigators may neglect quantitative analysis for interrelations between cases, contacts, and places. This could result in suboptimal selection of sampling objects and cases and my lead investigators to overlook infection propagation characteristics. [16, 17] Although some studies have focused on cases, [18–21] places, [22–24] and contact networks, [10, 25, 26] few studies have employed quantitative and graphics analyses, [15, 27] and no studies to date have combined SNA and GSUA approaches for quantitative and graphic analyses in field epidemiology for selecting sampling objects and characterizing infectious disease transmission in similar local outbreaks.

Nodes with high density or centrality are key to controlling and preventing disease outbreaks. [7, 8, 28] Selectively choosing only clinical symptoms may limit information regarding important vectors of transmission in those with subclinical or latent infections. [29, 30] SNA and GSUA enable quantitative methods for selection of sampling objects to help avoid loss of important patients. During the outbreak, 20 samples were selected in an unbalanced manner (17 from platoon A, only three from B), and the positive rate of the two rounds of sampling was poor (nine of 20 samples were positive). Therefore, the 14 nodes recommended by the SNA approach should be a priority for disease control, even in the absence of laboratory support. It should be noted that one limitation of this method is the inability to detect patients with latent infection; accordingly, we suggest collecting other samples.

The second problem pertained to mastering the characteristics of disease transmission. The strict security procedures of the detoxification center forbid all drug abstainers from bringing any electronic devices inside, including mobile phones, watches, and wearable sensors. We could also not obtain surveillance video data due to privacy considerations. Therefore, rather than state-of-the-art approaches such as SocioPatterns, [9, 20, 26, 31] we had to resort to direct and indirect individual data collection. Obtaining the data used to create Figures 2 and 3 required considerable investigation of three-dimensional distribution, contact information, and hospital data. In contrast, procuring the data used to create Figure 1 merely required collection of contact information. Accordingly, the SNA approach was more efficient with regards to grasping features of the disease outbreak. Furthermore, the government and the society paid a high attention on the outbreak, and all subjects investigated were able to recall details of the events, even 10 months later, during our retrospective investigation in May 2015.

The most important factor of our dynamic model was *k* (contact coefficient), which consistently presented a rapidly growing trend. Across the time span of the outbreak, we found a clear downward trend of *v* (infection coefficient), whereas *r* (recovery rate) played almost no role. Sums of the total indices different from a value of 1 indicate the presence of interactions among factors in the model.

In our study, 55 patients had been infected when the CDC was informed on July 20, 2014; four days after the first case fell ill. Thereafter, a total of 61 patients were infected until disinfection and quarantine on July 22. We also noted that until July 23, oseltamivir had been used for treatment and prevention purpose. Because the effective contacts among 96.83% of patients (61/63) were not influenced by disinfection, quarantine or treatment measures, the contribution of the contact coefficient increased continuously across the time span of the epidemic. This may explain why the change in sum of the first-order effects was relatively small.

Figure 4 was generated based on the guide of *Global Sensitivity Analysis: The Primer*. [6] Subsequently, the solution focused *via* transformation on *Y* (a concept similar to the basic reproductive rate, *R_0_*), where *Y = vk(S+I+R)*-*r*. In this case, *Y* > 0 indicates spread of the infection, and *Y* < 0 indicates subsidence of the infection. The *X* value (36, 425, 877) indicates the number of model runs (the total number of model runs was 1280) with *Y* being extremely close to zero (*Y* could be positive or negative), after which percentages were used to determine the degree of infection spread or subsidence. The infection propagated in 31.56% (1 - 877/1280 - 1/1280) of the cases in Figure 4, of which case 3 can be used as an example. Overall, if we wished to control the outbreak effectively and cause the infection rate to drop sharply, we would immediately perform the most stringent control measures, especially when dealing with highly infectious diseases in a relatively closed community.

In this study, data were collected primarily from interviews with affected patients; hence, the findings may be affected by recall-bias or non-response bias. Use of state-of-the-art methods would mitigate these biases to some degree. The conclusions of our study would also be more powerful and persuasive if it were possible to obtain virological data on all important nodes that selected by our approaches. [32, 33]

Previous studies have demonstrated that both the humoral and cellular immune systems are abnormal in drug abusers. [34, 35] Thus this population is theoretically more susceptible to influenza than the general population. However, all patients included in our study were male drug abstainers and their median age was 34 (interquartile range: 28 to 40), so that the discrepancy of age and gender would be equally true.

We suggest that SNA and GSUA approaches may be widely used for quantitative and graphic analyses on similar infectious disease outbreak and that additional prospective studies using molecular biological techniques be undertaken.

## MATERIALS AND METHODS

### Data sources

Data were collected from patients and staff members involved in the outbreak. We began by specifying the case definitions. Clinically diagnosed cases were defined as those with sudden onset of high fever, an axillary temperature of 38°C (100.4°F) or above, and at least three other clinical symptoms within the past week (dry cough, headache, muscle and joint pain, severe malaise, sore throat or runny nose). Laboratory-confirmed cases were defined as clinically diagnosed cases with influenza virus identified in respiratory specimens. Exclusion criteria included an axillary temperature below 38°C (100.4°F), fewer than three of the symptoms, negative laboratory test result before initiation of antiviral treatment, or no epidemic history. We also reinvestigated and recorded the time of onset (accurate to the hour), extent (direct contact defined as close contact within 1 m; indirect contact defined as touching objects that patients used), and place (dormitory, refectory, or other) of the close contacts of all 72 preliminary screening cases (index case: body temperature of 37.5°C (99.5°F) or above together with one of the symptoms, but without laboratory diagnosis). Here, we defined a pair of close contacts as two persons in contact irrespective of the time, extent, frequency, or place of contact. Secondly, we performed in-depth research on medical records, the field environment, disease control and prevention measures, and the managements regarding the outbreak. Finally, we interviewed all medical staff members and administrators to verify the information that had been previously collected.

This study was approved by the institutional review board of Tongji Medical College of Huazhong University of Science and Technology.

### Software utilized for data analysis

UCINET version 6.216 (Borgatti, S.P., Everett, M.G. and Freeman, L.C. 2002) was used to construct the matrix and calculate parameters for SNA. [36] NetDraw version 2.084 (Borgatti, S.P. 2002) was used to construct sociograms of the networks. [37] MATLAB version R2012a (Mathworks, Natick, MA, USA) was used to analyze the transmission through GSUA. [38] SAS version 9.4 (SAS Institute, Cary, NC, USA) was used to perform the chi-squared test, Cochran-Mantel-Haenszel statistic, and Kruskal-Wallis test. Statistical significance was set at *P* < 0.05.

### Data processing

We collected contact information from all 72 index cases and established a matrix referred to as “whole72”. We calculated the degree centrality and betweenness centrality of all index cases (Table 1). Comparison of degree and betweenness centrality between traditional method and SNA approach was analyzed using the Kruskal-Wallis test (Tables 2 and 3).

Based on clinical diagnosis criteria, laboratory test results and epidemic history, nine nodes were excluded (see Table 1), and 63 nodes were recognized as patients who then formed the matrix “adjust63”. We then added two types of relationships (platoon A, platoon B) to create a new matrix, “adjust65”. We generated a structure and distribution diagram of outbreak networks (Figure 1). Based on onset time, contact time and spatial distribution (dormitory), we drew two diagrams of the possible transmission pattern (Figures 2 and 3).

Parameter *I* represented the number of infected individuals at time *t*, parameter *S* represented the number of individuals susceptible to infection at time *t*, and parameter *R* represented the number of recovered individuals at time ***t***. Factors *v* and *r* represented the “infection coefficient” and “recovery rate”, respectively, factors *v* and *r* were both in accord with a normal distribution. Factor represented the “contact coefficient”, which was distributed as (2, 7) at the beginning of the influenza outbreak, as ***k ~ beta*** (0.5, 10) during the period of quarantine, and as ***k ~ beta*** (0.2, 15) when all patients and susceptible individuals had received oseltamivir. The dynamic equations of our retrospective study are

$$\begin{array}{ccc} \frac{dl}{dt} & = & vkIS\;-\;rI\\ \frac{dS}{dt} & = & -vkIS\\ \frac{dR}{dt} & = & rI \end{array}$$

We calculated sensitivity indices for the three factors. Results for the three configurations of ***k*** were shown in Table 4. We then performed uncertainty analysis with the model simulation outputs (Figure 4).
